# ^1^H-detected characterization of carbon–carbon networks in highly flexible protonated biomolecules using MAS NMR

**DOI:** 10.1007/s10858-023-00415-6

**Published:** 2023-06-08

**Authors:** Salima Bahri, Adil Safeer, Agnes Adler, Hanneke Smedes, Hugo van Ingen, Marc Baldus

**Affiliations:** NMR Spectroscopy, Bijvoet Center for Biomolecular Research, Utrecht University, Padualaan 8, 3584 CH Utrecht, The Netherlands

**Keywords:** Magic Angle Spinning, Protein dynamics, Tau, Microtubules, Fungal cell wall

## Abstract

In the last three decades, the scope of solid-state NMR has expanded to exploring complex biomolecules, from large protein assemblies to intact cells at atomic-level resolution. This diversity in macromolecules frequently features highly flexible components whose insoluble environment precludes the use of solution NMR to study their structure and interactions. While High-resolution Magic-Angle Spinning (HR-MAS) probes offer the capacity for gradient-based ^1^H-detected spectroscopy in solids, such probes are not commonly used for routine MAS NMR experiments. As a result, most exploration of the flexible regime entails either ^13^C-detected experiments, the use of partially perdeuterated systems, or ultra-fast MAS. Here we explore proton-detected pulse schemes probing through-bond ^13^C–^13^C networks to study mobile protein sidechains as well as polysaccharides in a broadband manner. We demonstrate the use of such schemes to study a mixture of microtubule-associated protein (MAP) tau and human microtubules (MTs), and the cell wall of the fungus *Schizophyllum commune* using 2D and 3D spectroscopy, to show its viability for obtaining unambiguous correlations using standard fast-spinning MAS probes at high and ultra-high magnetic fields.

**Supplementary Information:**

The online version contains supplementary material available at 10.1007/s10858-023-00415-6.

## Introduction

Magic Angle Spinning (MAS) NMR is a powerful technique to study the structure, dynamics, and intermolecular interactions of insoluble biomolecules and materials. Over the last three decades, the nature of biomolecules that can be studied with MAS NMR have become more complex, from short peptides (Fu and Cross 1999; Griffin 1998; Jaroniec et al. 2002; Luca et al. 2003; Rienstra et al. 2000; Tycko 2001) to whole cells (Ghassemi et al. 2021; Narasimhan et al. 2019; Renault et al. 2012). Concomitant technological advances have brought fast- to ultra-fast spinning probes (Barbet-Massin et al. 2014; Penzel et al. 2019; Samoson 2021) for ^1^H-detection using progressively smaller rotors, which reduce linewidths in the case of homogeneous broadening (Maricq and Waugh 1979; Schledorn et al. 2020) while maintaining practical sensitivity due to the relatively large ^1^H gyromagnetic ratio (Agarwal et al. 2014). Such advances have led to the development of MAS ^1^H-detection techniques of fully protonated samples, circumventing the expense and loss of information of sample deuteration (Guo et al. 2014; Paulson et al. 2003; Zhou et al. 2007, 2012). As a result, a suite of ^1^H-detected experiments for de novo chemical shift assignments (Barbet-Massin et al. 2014; Fricke et al. 2017; Stanek et al. 2016) are increasingly commonplace and include frequent use of dipolar or scalar ^13^C–^13^C mixing schemes such as RFDR (Asami and Reif 2013; Bennett et al. 1992; Paluch et al. 2022; Sarkar et al. 2022), TOBSY (Baldus et al. 1997; Baldus and Meier 1996), WALTZ (Stanek et al. 2016), and DIPSI (Paluch et al. 2022; Shaka et al. 1988). Typical uses for these mixing techniques have occurred within the rigid part of biomolecular assemblies, as selected by cross-polarization at the start of the pulse sequence (Barbet-Massin et al. 2013).

However, for insoluble systems comprising flexible components, INEPT transfers select for nuclei in the fast-motion regime where dipolar self-averaging occurs. The INEPT-TOBSY experiment is frequently used to map ^13^C–^13^C connectivities in conjunction with dipolar-based counterparts, typically in a ^13^C-detected fashion (Andronesi et al. 2005; Baldus and Meier 1996; Damman et al. 2019; Heise et al. 2005). Heteronuclear ^1^H–^13^C-detected correlations can provide additional assignments for the flexible regime (Alam and Holland 2006; Damman et al. 2019; Elena et al. 2005; Siemer 2020). In the case of limited sample quantities, it is instead desirable to amplify the signal using ^1^H-detection, which requires an INEPT transfer back to the ^1^H channel following ^13^C evolution, mixing, and water suppression (Zhou and Rienstra 2008). Variants of a scalar 2D hCH *J*-HSQC experiment have been used to characterize complex systems including fungal cell walls (Safeer et al. 2023), human microtubules (Luo et al. 2020; Savastano et al. 2020), viral capsids (Callon et al. 2022), protein condensates (Damman et al. 2019) and membrane-bound proteins (Howarth and McDermott 2022). In addition, INEPT based homonuclear 1-bond transfers have been used to obtain sequential backbone correlations in highly mobile amyloid-fibrils (Falk and Siemer 2016; Zhang et al. 2023).

In the following, we explore the use of ^1^H detected experiments that involve an additional broadband through-bond CC transfer step to increase spectral dispersion in applications on complex biomolecular systems containing dynamic molecular (sub)species that are difficult to prepare in deuterated form. Such an INEPT-based 3D hCCH through-bond sequence with longitudinal mixing has previously been used at moderate spinning (20 kHz) on deuterated samples recrystallized in 90% D_2_O, to obtain residual protonated methyl group assignments while avoiding ^1^H_2_O suppression (Agarwal and Reif 2008). In addition, a 2D hC(c)H TOCSY experiment with DIPSI-2 mixing was used to obtain highly resolved spectra on a fully protonated peptidoglycan with an ultra-fast spinning probe (Bougault et al. 2019). However, in our hands, such usage of longitudinal homonuclear mixing is prone to producing antiphase artifacts generated by resonances with especially long coherence lifetimes. We found that these artifacts are resolved either by using long phase cycles that render extension to a 3D experiment impractical, or by instead using transverse mixing with a prior trim pulse (Bax et al. 1990) to purge unwanted coherences, similarly implemented as in solution-state NMR. We apply these schemes to study the flexible region of two complex insoluble systems using hCCH TOCSY, with DIPSI-3 (Shaka et al. 1988) and WALTZ (Shaka et al. 1983) homonuclear carbon mixing in gradient-free MAS probes. We demonstrate that such schemes provide broadband ^13^C–^13^C correlations at both high and ultra-high field NMR conditions.

## Material and methods

### Expression of tau K32

The gene encoding tau K32 inserted in a pNG2 plasmid was kindly gifted to us by the Mandelkow group of the German Center for Neurodegenerative Diseases, Bonn, Germany. The vector was transfected in *E. coli BL21 (DE3)* competent cells and expression was induced in M9 minimal media with 0.3 mM isopropyl–D-thiogalactopyranoside (IPTG) when the OD_600_ reached 0.6 for 16 h at 20 °C.

### Purification of tau K32

[^13^C,^15^N] labeled tau K32 was purified according to the protocol described by Barghorn et al. (2005). Small adaptations to the protocol are described below. Firstly, 1 mM NaN_3_ was added to the buffers, to minimize the risk of growth in them. Moreover, cells were lysed either with a French pressure cell as described previously or by sonication. The cation exchange chromatography that was utilized was a HiTrap SP column of 5 mL and for gel filtration chromatography a HiLoad 26/60 Superdex 75 prep grade column. The protein concentration was measured by a BCA assay with a Thermo Scientific Pierce BCA Protein Assay Kit.

### MT polymerization and preparation of solid-state NMR samples

Lyophilized tubulin (Cytoskeleton, Inc.) was solubilised in Brb80 buffer (80 mM PIPES, 2 mM MgCl2, 1 mM EGTA, pH 6.8, 1 mM NaN3, 1 mM DTT, pH 6.8), to a final concentration of 2 mg/mL. The polymerization was induced by adding 1 mM Guanosine-5′-triphosphate (GTP) and incubation for 15 min at 30 °C. Then, 20 μM paclitaxel (taxol, SIGMA) was used to stabilize the MT and incubation took place for another 15 min at 30 °C. The MT were spun down at 180.000 × *g* (Beckman TLA-55 rotor) for 30 min at 30 °C and the pellet was resuspended in warm Brb80 buffer with 20 μM paclitaxel. Subsequently, a 1:1 ratio of ^13^C^15^N tau K32 was added. The interaction partners were incubated for 30 min at 37 °C. In the following isotopically labelled tau K32 in complex with MT was separated from the unbound, non-polymerised fraction by centrifugation at 180.000 xg (Beckman TLA-55 rotor) for 30 min at 30 °C. Afterwards, the pellet was washed with 40 mM phosphate buffer, pH 7, with protease inhibitor (as described earlier) and 1 mM NaN3, without disturbing the pellet. A 1.3 mm rotor was packed with the pellet.

Basic experimental details for each sample used in this manuscript. Further details are provided in the materials and methods section, and Table S1.

### MAS NMR experiments

**Fig. 1:**
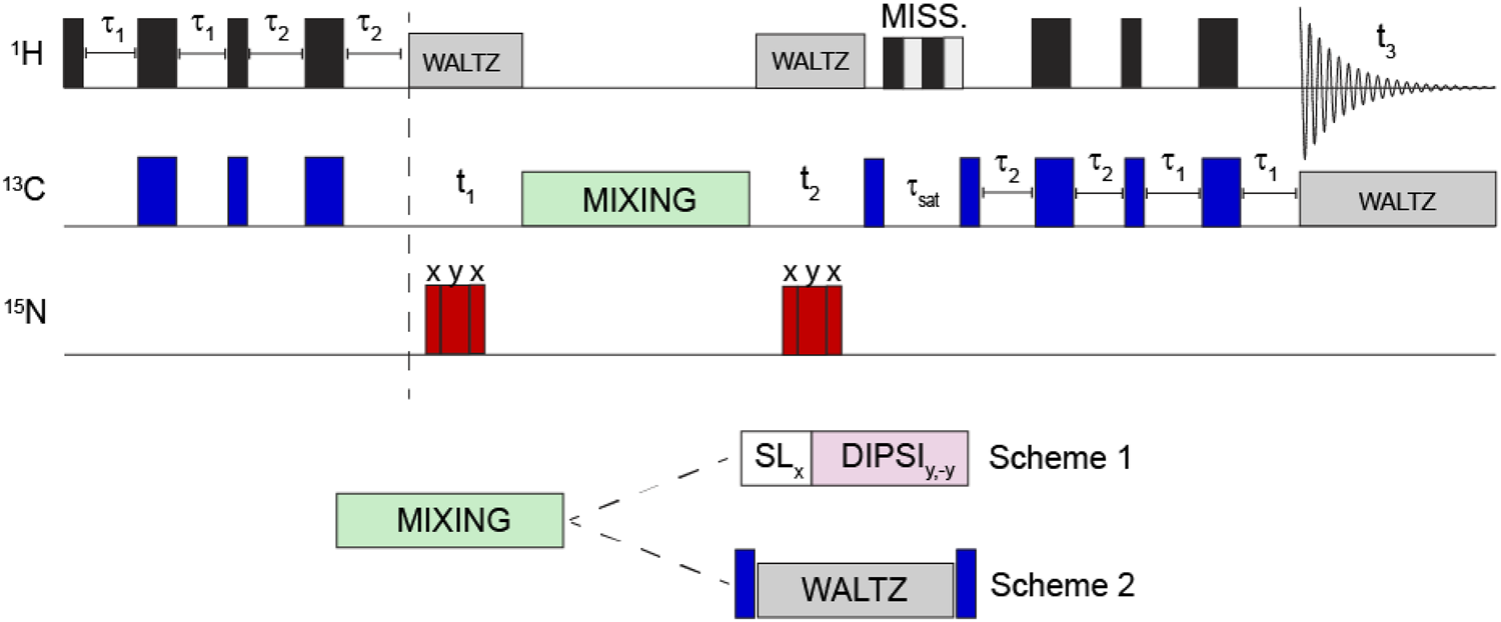
hCCH 3D used in this work. First, an INEPT block transfers magnetization through-bond from ^1^H to ^13^C prior to an evolution period to encode the 13C chemical shift. After the t_1_ period, mixing is applied according to Scheme 1 or Scheme 2. Afterwards, the chemical shift is again encoded during the t_2_ period. The carbon magnetization is then stored longitudinally during the water suppression period, prior to INEPT transfer back to protons for detection. Narrow and wide rectangles indicate 90°/180° pulses, respectively. The 3 hC(c)H experiments were collected by not evolving the t_2_ period. Scheme 1 was used for the tau-microtubule complex, while Scheme 2 was used for *S. commune*. More details regarding the experiments are available in the Supplementary information

MAS NMR experiments for the tau-microtubule mixture used a 3-channel HXY 1.3 mm probe at ω_0H_/2π = 700 MHz with a Bruker Avance 3 console. The MAS rate was set to 44 kHz, with a set temperature of 280 K, which results in a sample temperature of ~303 K based on calibration using KBr (Thurber and Tycko 2009). The pulse program corresponding to this sample is provided schematically in Fig. S1, with the full sequence in the supporting information. Pulse amplitudes for INEPT transfers and ^15^N *J*-refocusing were the following: ω_1H_/2π = 127 kHz, ω_13C_/2π = 83 kHz, and ω_15N_/2π = 75 kHz. MISSISSIPPI was used for water suppression (Zhou and Rienstra 2008). 2D experiments were collected by not evolving the t_2_ dimension of the pulse scheme shown in Fig. 1. For 0 ms mixing, neither the SL_x_ nor the DIPSI_y,-y_ blocks were executed, while in the case of mixing we used 2 ms of SLx at the same amplitude as the DIPSI strength (Bax et al. 1990; Clore et al. 1990). The 2D experiments were processed in Topspin: the data was zero-filled, and a QSine window function was applied with a sinebell shift of 2.5. For the 3D, non-uniform sampling was used with a Poisson Gap distribution schedule with 25% sampling density (Maciejewski et al. 2012), out to ~9 ms of acquisition time in both indirect ^13^C dimensions. The data was then reconstructed using the SMILE NUS reconstruction algorithm (Ying et al. 2017) in NMRBox (Maciejewski et al. 2017) using NMRPipe (Delaglio et al. 1995) for preparatory and post-processing. Each dimension was zero-filled, and sine bell-squared window processing was used for both indirect dimensions, with an offset of 0.5, endpoint of 0.95, and exponent 1.0. For SMILE processing, the noise factor for signal cutoff was set to 5, with an 80% threshold for signal detection. In both the 2D and 3D experiments, the ^1^H dimension was referenced using water at 4.7 ppm, while the ^13^C dimension was referenced indirectly with a correction factor determined from the gyromagnetic ratio. Chemical shift and linewidth analysis was performed in NMRFAM Sparky software (Lee et al. 2015).

The ^13^C/^15^N *S. commune* sample was prepared as discussed in previous publications (Ehren et al. 2020; Safeer et al. 2023). MAS NMR experiments used a 3-channel HXY 1.3 mm probe at ω_0H_/2π = 1.2 GHz with a Bruker NEO console. The MAS spin rate was set to 60 kHz, and the sample was cooled to 260 K leading to a sample temperature of ~20 °C. The pulse sequence outlined in Fig. S1b was used. The strength of the hard pulses on each channel were set as follows: ω_1H_/2π = 100 kHz, ω_13C_/2π = 67 kHz, and ω_15N_/2π = 50 kHz. The ^1^H dimension was referenced using water referenced to 4.7 ppm, while the ^13^C dimension was referenced to previously published work (Ehren et al. 2020). B_0_ field drift artifacts in the *S. commune* spectra were corrected using previously published scripts (Najbauer et al. 2019). Further information regarding data acquisition for both samples can be found in Table 1 and Table S1.

**Table 1:** Summary of MAS NMR experimental parameters used in this work

Sample	Tau + MT	S. commune
$\omega _{0H} /2\pi$	700 MHz	1200 MHz
$\omega_{r} /2\pi$	44 kHz	60 kHz
Sequence scheme	Scheme 1	Scheme 2
$\tau_{1}$	1.72 ms $(1/4{\text{J}}_{HC} )$	1.72 ms $(1/4{\text{J}}_{HC} )$
$\tau_{2}$	1.15 ms (1/6J_*HC*_)	1.72 ms $(1/4{\text{J}}_{HC} )$
^13^C–^13^C Mixing, ω_13c_/2π	SL_x_ + DIPSI, 17 kHz	WALTZ-16, 15 kHz

## Results and discussion

**Fig. 2:**
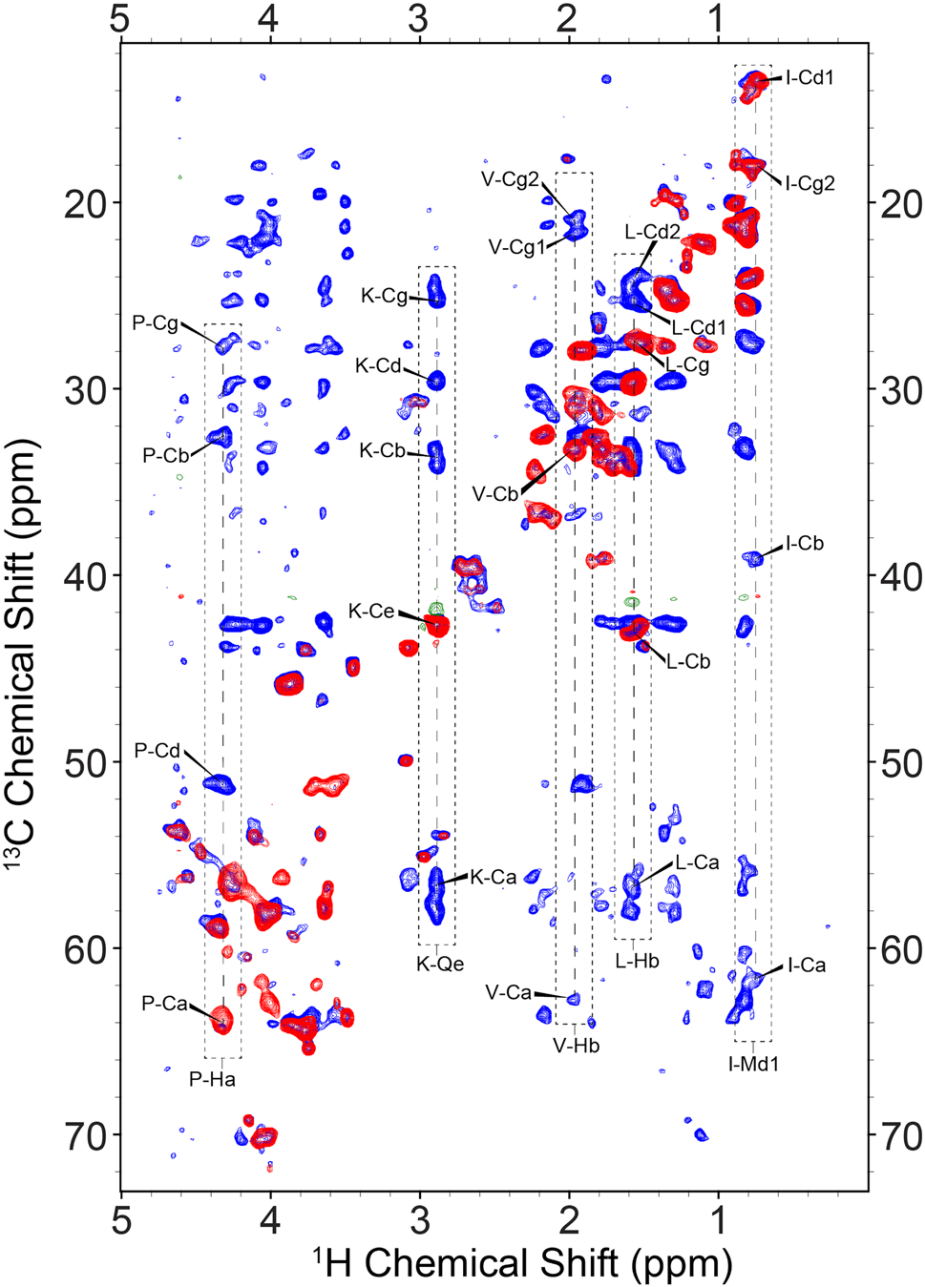
2D hC(c)H spectral overlay of 0ms (red/green) and 25ms (blue/orange) of DIPSI mixing for the tau-microtubule complex at 700 MHz (^1^H Larmor frequency). In the dashed boxes we show examples of cross-peaks corresponding to complete intraresidual (along the side chain) scalar transfers within various residues, with unambiguous correlations as determined in the 3D shown in Fig. 3. In each vertical strip, the peaks in red are the resonance from which the first ^1^H→^13^C transfer originates before transferring to the blue crosspeaks

In the following, we explore the flexible regions of two complex insoluble systems using hCCH TOCSY, with broadband DIPSI-3 (Shaka et al. 1988) and WALTZ-16 (Shaka et al. 1983) homonuclear carbon mixing in gradient-free probes using the pulse schemes given in Fig. 1. We tested pulse Scheme 1 in Fig. 1 on a sample of [^13^C,^15^N] labeled tau in complex with unlabeled microtubules (MTs). Tau is an intrinsically disordered protein that maintains microtubule stability (Brotzakis et al. 2021; Kadavath et al. 2015), and whose function is modulated by a series of posttranslational modifications including phosphorylation, nitration, and glycosylation (Martin et al. 2011). In the current context we used the K32 variant (~20.5 kDa of the full length), corresponding to the microtubule binding domain Q244-K369 (R1, R2, R3, R4), the N-terminal proline-rich region S198-Q244 (P2), and the C-terminal R’ region K369–Y394 (Brotzakis et al. 2021). For labeled tau bound to MTs, application of the 2D hC(c)H experiment shown in Fig. 1 Scheme 1 with 2 ms of preparatory *C*_*x*_ spin lock in addition to 25 ms of DIPSI-3 mixing, revealed multiple-bond CH correlations, to assign the protein backbone and non-aromatic sidechains (Fig. 2, blue). For reference, a 2D spectrum with 0 ms of mixing is shown in red.

**Fig. 3:**
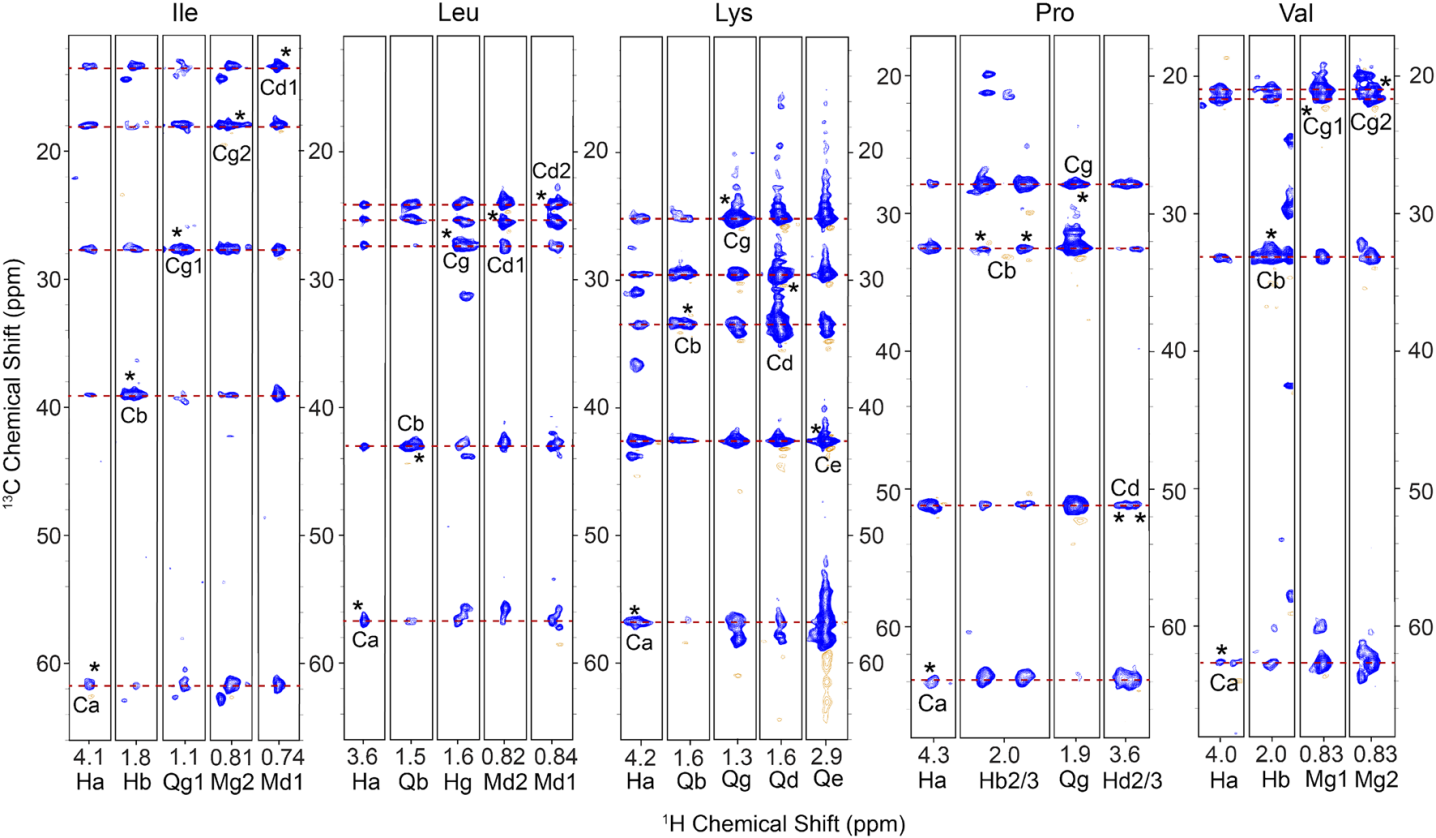
Strips from a 3D hCCH TOCSY lengths spectrum of the tau-microtubule mixture at 700MHz using 25ms of DIPSI mixing, in which we show correlations from the backbone and sidechain of four example residues: Isoleucine, Leucine, Lysine, Proline and Valine. Asterisks indicate peaks along the diagonal of the CC plane. For Proline, we observe doubled peaks for protons originating from Cb/Cd diagonal peaks

In line with previous work (El Mammeri et al. 2022) we expect that the observed correlations result from flexible tau residues not involved in strong binding to MTs. While typical chemical shift assignments allow for immediate recognition of some of these resonances (e.g. Ile), chemical shift degeneracy results in a heavy degree of spectral overlap, which was alleviated by adding a second dimension to the experiment after the DIPSI-3 mixing period. Figure 3 shows the resulting strips corresponding to Ile, Leu, Lys, Pro, and Val sidechains. We also identified additional Ile and Lys residues in the 3D spectrum (not shown), indicating its usefulness in distinguishing like residues whose chemical shift dispersion results in significant overlap in lower dimensional experiments. Finally, we observe several distinct Ala, Thr and Gly residues (not shown). Currently, our survey of identifiable residues is in agreement with those observed previously (El Mammeri et al. 2022), though we find that our 3D hCCH correlations lead to more complete chemical shift assignments from backbone and sidechain resonances. Amino-acid selective assignments and proton linewidths for the residues highlighted in Fig. 3 are provided in Table S2. While the average linewidth is less than 0.1 ppm, the observed variations did not correlate with backbone versus sidechain topology or degree of protonation.

Next, we tested the suitability of INEPT-based ^13^C–^13^C scalar transfers to explore the dynamic region of the cell wall of *S. commune*. Previous studies of ^13^C/^15^N-labeled *S. commune* reveal that it is composed of a complex mixture of proteins and various kinds of polysaccharides(Ehren et al. 2020; Safeer et al. 2023). Consequentially, the carbon spectrum features a larger protonated ^13^C chemical shift dispersion (~100 ppm) while the non-aromatic ^1^H shift range remains small (0–6 ppm). As a result, low-power irradiation for homonuclear CC transfer leads to shorter-range transfer, especially at ultra-high field.

**Fig. 4:**
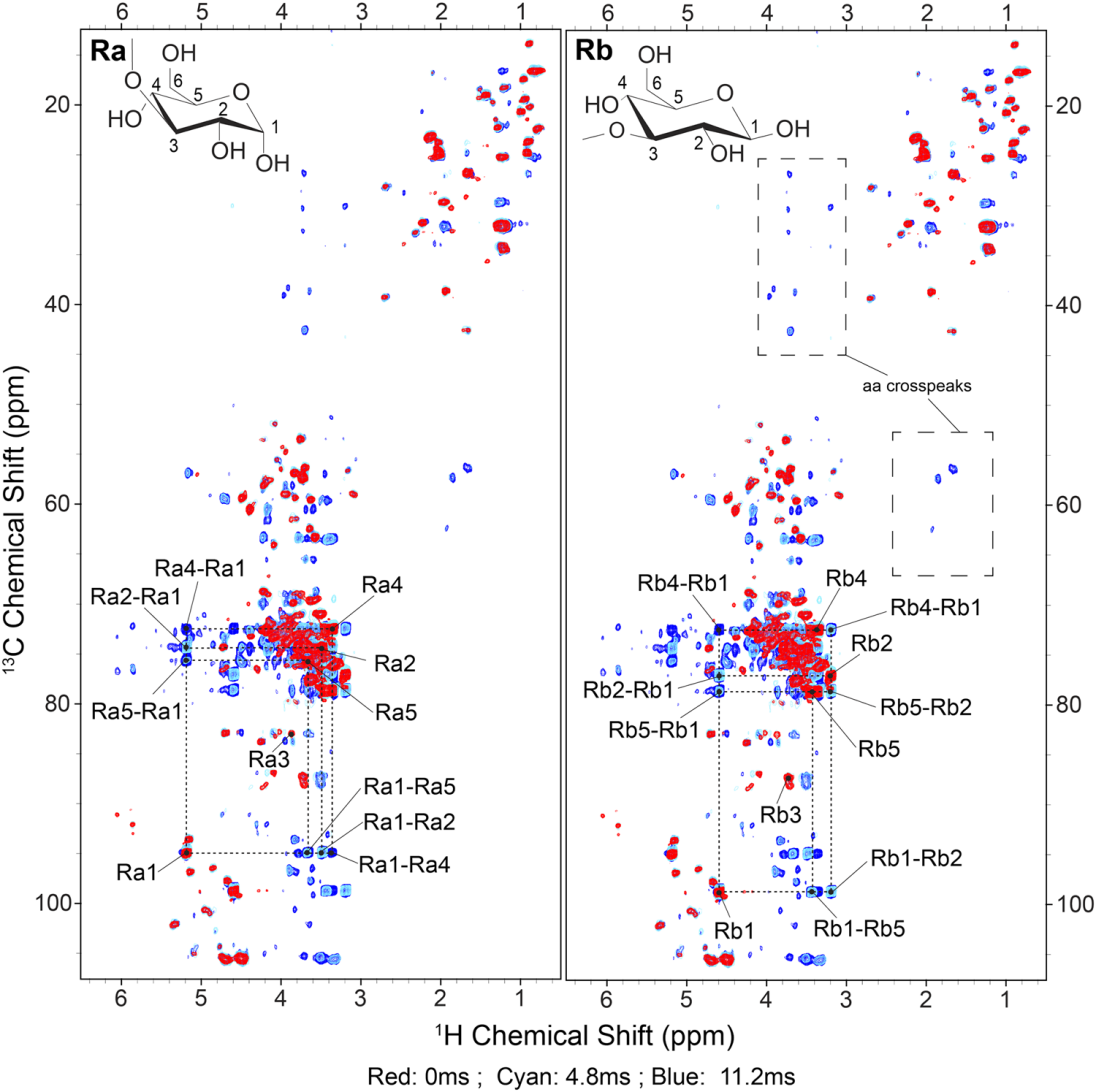
Comparing hC(c)H spectra of *S. commune* with varying TOCSY lengths, using WALTZ-16 at 1200 MHz (^1^H Larmor frequency). We specifically show peaks belonging to the reducing ends of -glucan (Ra, left) and -glucan (Rb, right) as they are relatively well-isolated and show prominent transfer. In red we show the spectrum with no mixing, so that all peaks correspond to directly bonded C–H pairs. The cyan (4.8 ms) and blue (11.2 ms) spectra instead show multiple bond correlations corresponding to various combinations of C–H pairs within the pyranose ring (structure shown). We also observe correlations among the amino acid region (dashed boxes). For fungal cell wall biological component analysis of 0 vs. 11.2 ms data, see Safeer et al. 2023

For this system, we used a variant of the sequence as shown in Fig. 1 Scheme 2, in which τ_1_ = τ_2_ = 1/(4*J*_HC_), favoring the CH groups from polysaccharides and amino acids to alleviate spectral congestion. Figure 4 shows overlays of 3 spectra with 0 ms (red), 4.8 ms (cyan), and 11.2 ms (blue) of isotropic WALTZ-16 mixing, which has a larger bandwidth than DIPSI-3 and may thus be advantageous at ultra-high field (Shaka et al. 1988). In spite of these relatively short mixing times, low-power irradiation (15 kHz) resulted in efficient transfers among nuclei within the polysaccharide region (~ 65–105 ppm). We highlight unambiguous assignments corresponding to the reducing ends of α- (**Ra**, left) and β-glucan (**Rb**, right), which are both formed from a pyranose ring in chair conformation, in which C3 links the ring to C1 of the successive sugar subunit via a glycosidic bond. We observe nearly all correlations from the pyranose ring with 11.2 ms of mixing, with the exception of C3 and C6; the former is observed only weakly in INEPT-based experiments due to its reduced mobility, while the latter has been filtered out by our choice in τ_2_ INEPT delays. Notably, we also observe weak correlations between the amino acid backbone and sidechain resonances at 11.2 ms of mixing (Fig. 4, right). As shown for K32-tau, stronger correlations in this region may require longer mixing for complete transfer. Higher transfer efficiency might also be attained with the use of DIPSI instead of WALTZ mixing, which has higher transfer efficiency though at the cost of a narrower bandwidth (Shaka et al. 1988).

Notably, we found little overlap between peaks that appear in the INEPT-based spectrum in Fig. 4 and a CP-based hCH correlation experiment of the same sample of *S. commune* (Safeer et al. 2023). We thus expect that all observable correlations arise from the flexible domain of the fungal cell wall where chemical shift anisotropies and dipolar couplings are averaged out. Interestingly, when comparing the ^1^H linewidths between the tau 3D spectra (Table S1) and fungal 2Ds (Safeer et al. 2023), we observe slightly broader average ^1^H linewidths for the tau data (~0.093 ppm), which was collected at 44 kHz MAS vs. 60 kHz MAS for *S. commune* (~0.067 ppm). This improvement may be attributed to the nature of the sample or differences in effective temperature, and the larger magnetic field in the case of *S. commune*.

## Conclusions

In this work we have introduced two and three-dimensional ^13^C–^13^C–^1^H correlation experiments that allowed us to characterize dynamic carbon networks of complex biomolecular systems without the need of deuteration, HR-MAS probes (Blicharski and Sobol 1982; Howarth and McDermott 2022; Li et al. 2005; Zupancic and Pirs 1976) or ultra-fast MAS in ^1^H-detected TOCSY experiments(Ikura et al. 1990; Kay et al. 1990). Our data show that we can indirectly determine through-bond ^13^C–^13^C connectivities with refocused INEPT-based pulse sequences in a selective manner as determined by the choice of *J*-coupling and delays. We obtained 2D hC(c)H spectra of exceptionally high resolution on a fully protonated mixture of ^13^C/^15^N-labeled K32-tau and unlabeled human microtubules as well as fungal cell wall preparations of *Schizophyllum commune*(Ehren et al. 2020). We extended the sequence to a 3D hCCH experiment to alleviate chemical shift degeneracy for obtaining unambiguous correlations. This experiment could be further tuned to additional selectivity, by adjusting the length of the τ_2_ delay. Combination with ^1^H-detected hNCOCA and hNCA experiments (Falk and Siemer 2016; Linser et al. 2008, 2010; Zhang et al. 2023) would lead to full sequential assignments of dynamic protein stretches. The hCCH experiment could also be expanded to a 4-dimensional hNCACH sequence, in which the N-edited transfer to CA’s selects for backbone resonances, expanding the information available in a single experiment.

This method can also be used in conjunction with CP-based ^1^H-detected schemes—which require fast-to-ultrafast MAS techniques for fully protonated samples—and also enable one to study biopolymers within native conditions. Adapting solution NMR methodology in this way will allow Magic Angle Spinning NMR to explore a wider scope of biomolecules with a large flexible domain, such as membrane proteins whose main functions lie in the extra membrane space e.g. integrins (Bergonzini et al. 2022), growth hormone receptors (Kaplan et al. 2016), and membrane-anchored lipoproteins (Xiang et al. 2022). In addition, such analysis is not restricted to biomolecules, but could also be used to identify soluble organic intermediates and byproducts in industrial catalysts (Chowdhury et al. 2018a, b). We thus predict widespread usage of such sequences using standard MAS probes to thoroughly characterize organic components across various applications.

## Supplementary Information

Below is the link to the electronic supplementary material.

Supplementary file1 (DOCX 737 KB)
