# Supplementary material for: 1H-detected characterization of carbon–carbon networks in highly flexible protonated biomolecules using MAS NMR
Source: J Biomol NMR. 2023 Jun 8;77(3):111–9. doi: 10.1007/s10858-023-00415-6 (PMC10307723; doi:10.1007/s10858-023-00415-6)
Supplement: Supplementary file 1 — Supplementary file1 (DOCX 737 KB) [file 10858_2023_415_MOESM1_ESM.docx]

**Supplementary Information**

**^1^H-detected characterization of carbon-carbon networks in highly flexible protonated biomolecules using MAS NMR**

Salima Bahri*, Adil Safeer, Agnes Adler, Haneke Smedes, Hugo van Ingen, and Marc Baldus*

1. Scheme 1


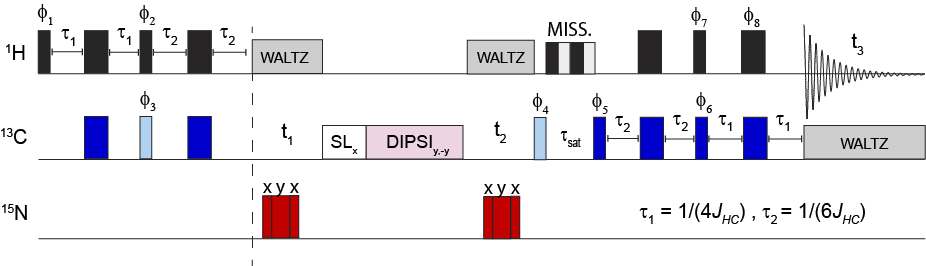


1. Scheme 2


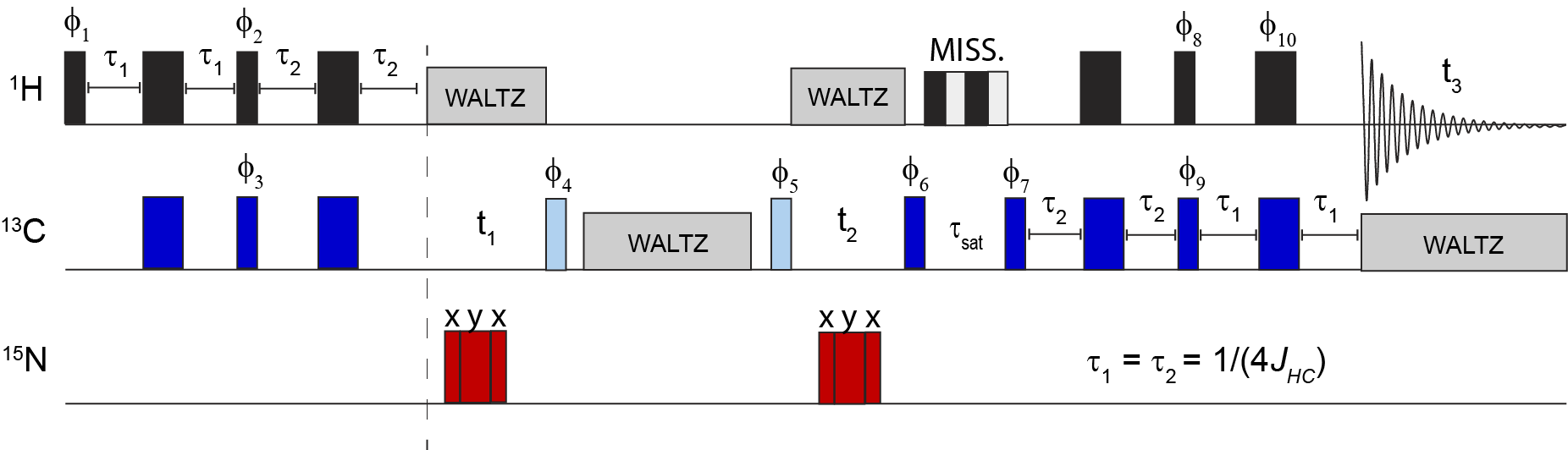


**Fig. S1** (a) The phase cycle was set as follows: φ_1_= *x*, φ_2_ = -*y*, φ_3_ = *x -x*, φ_4_ = *y*, φ_5_ = *-y -y y y*, φ6 = *-x*, φ_7_ = *x*4 -x*4*, φ_8_ = *x -x*, φ_rec_ = *x -x -x x -x x x -x*. The phases of the ^15^N pulse were *x y x* as indicated. Unless otherwise stated, the pulse phase was set to *x*. In light blue we show the pulses that are independently phase-shifted for quadrature detection. (b) The phase cycle was set as follows: ; φ_1_= *x -x*, φ_2_ = -*y*8 y*8*, φ_3_ = *x*16 -x*16*, φ_4_ = *y*, φ_5_ = *-y*32 y*32*, φ_6_ = *y*4 -y*4*, φ_7_ = *y*, φ_8_ = *x x -x -x*, φ_9_ = *-x*, φ_10_ = *x -x*, φ_rec_ = *x -x -x x -x x x -x -x x x -x x -x -x x -x x x -x x -x -x x x -x -x x-x x x -x.* In light blue we show incremented pulses for States-TPPI


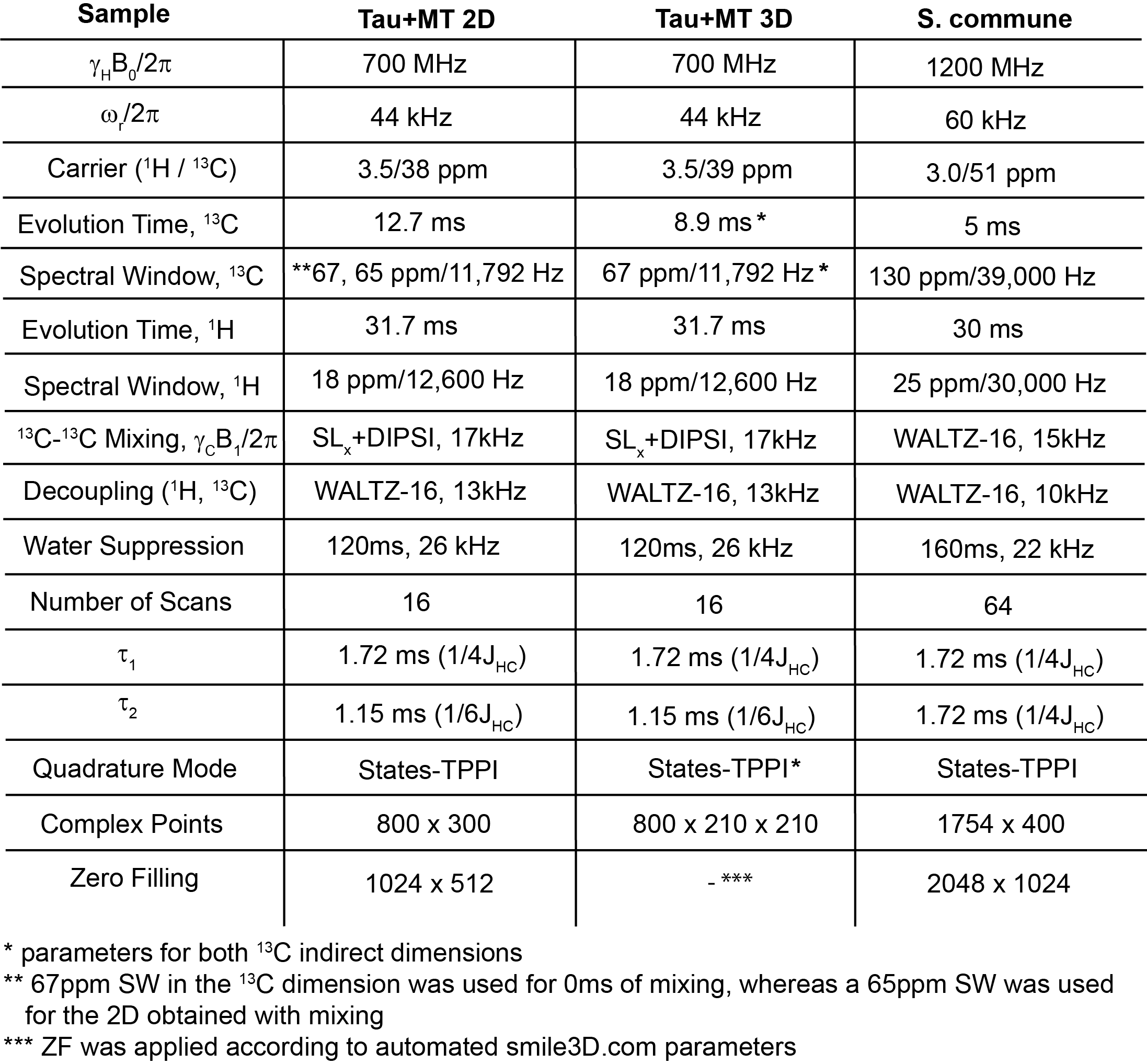


**Table S1** Acquisition/processing parameters for the Tau+MT mixture and *S. commune*.


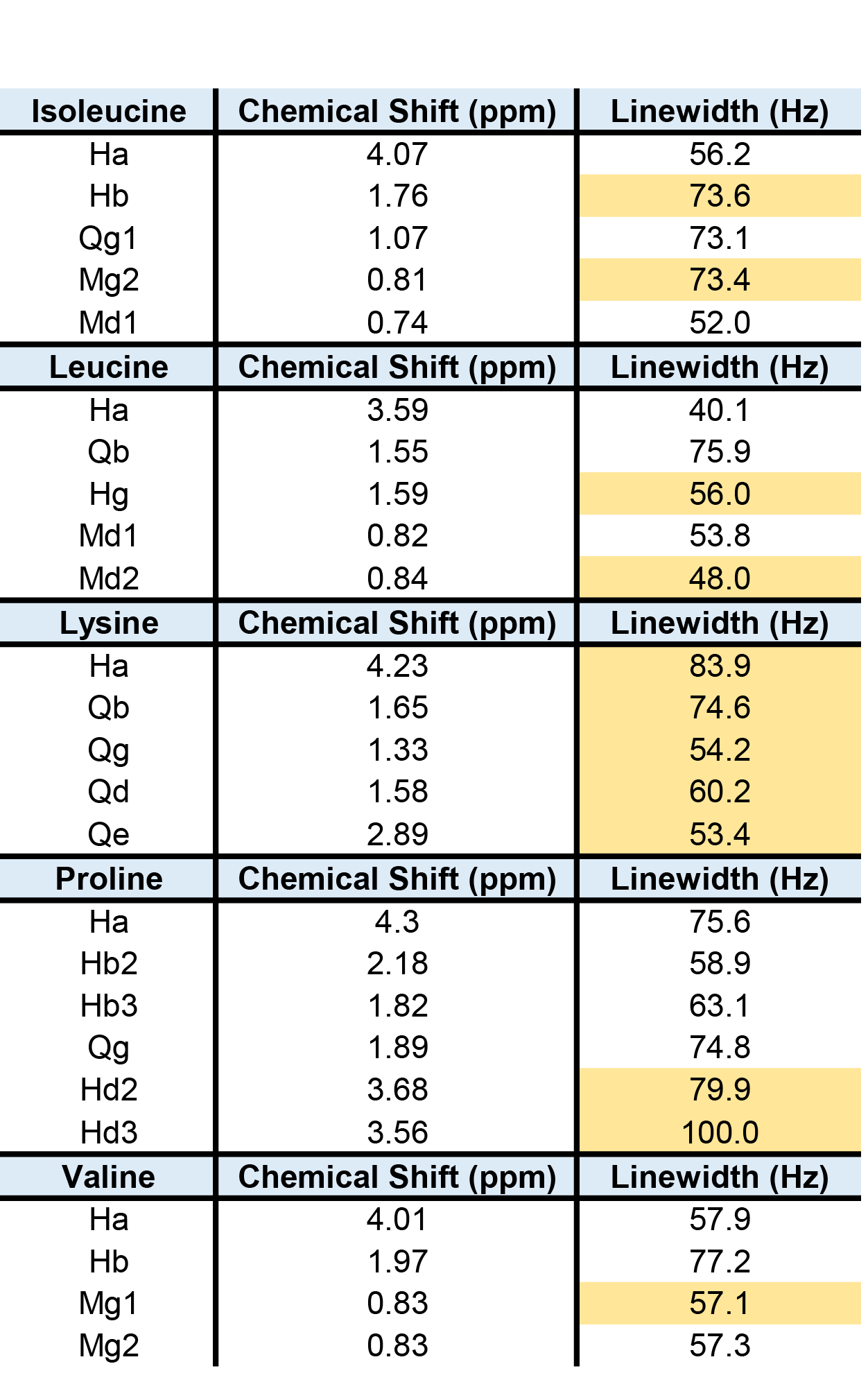


**Table S2** ^1^H chemical shifts and linewidths of tau resonances (Fig. 3 as determined in Sparky. Unresolved peaks are highlighted in yellow. Linewidths were measured in the directly bonded CH plane, in the 3D slice corresponding to the diagonal CC peak.

**Pulse Sequence, Bruker Format**

;$COMMENT= hCCH 3D, arbitrary contact and decoupling schemes

;$CLASS=Solids

;$DIM=3D

;$TYPE=INEPT

;$OWNER=Bruker

;p1 : H90 pulse length

;p2 : C90 pulse length

;p3 : N90 pulse length

;p20 : MISSISSIPPI pulse length

;p9 : f2 channel - 90 degree low power pulse (DIPSI)

;pl1 : H90 pulse power

;pl2 : C90 pulse power

;pl3 : N90 pulse power

;pl13 : H WALTZ decoupling

;pl29 : C WALTZ decoupling

;pl14 : C DIPSI3 power

;cnst2 : H-C J-coupling

;cnst24 : frequency hop for water suppression

;cnst3 : relection for echo, 6 to select all 13C

;l1 : to set length of DIPSI mixing

;l2 : MISSISSIPPI loops

define delay JCH1

define delay JCH2

define delay JCH2t

define delay mix

"d0 = 0"

"l0 = 0"

"d10 = 0"

"l10 = 0"

"in0 = inf1"

"in10 = inf2"

"p5 = 2000u"

"d16 = 0.636*p1"

"mix=(p9*54.33*4)*l1"

"JCH1 = 1/(4*cnst2)"

"JCH2 = 1/(cnst3*cnst2)"

"JCH2t = JCH2 - 0.636*p2"

1 ze

2 d1 do:f2 do:f1

(p1 pl1 ph1):f1

JCH1

(center (p1*2 pl1 ph0):f1 (p2*2 pl2 ph0):f2)

JCH1

(center (p1 pl1 ph2):f1 (p2 pl2 ph3):f2)

JCH2t

(center (p1*2 pl1 ph0):f1 (p2*2 pl2 ph0):f2)

JCH2 pl13:f1 pl3:f3

if "l0 == 1" {

"d0 = in0 - 0.3u"

}

if "l0 > 0" {

(center (d0 cpds1):f1 (p3 ph20 p3*2 ph21 p3 ph20):f3)

0.3u do:f1

}

#ifdef tocsy ; note for no mixing, remove tocsy flag

(p5 pl5 ph0):f2

0.3u pl14:f2

9 (p9*2.722 ph21):f2

(p9*4.389 ph23):f2

(p9*2.778 ph21):f2

(p9*3.056 ph23):f2

(p9*0.333 ph21):f2

(p9*2.556 ph23):f2

(p9*4.000 ph21):f2

(p9*2.722 ph23):f2

(p9*4.111 ph21):f2

(p9*3.778 ph23):f2

(p9*3.889 ph21):f2

(p9*2.889 ph23):f2

(p9*3.000 ph21):f2

(p9*0.333 ph23):f2

(p9*2.500 ph21):f2

(p9*4.050 ph23):f2

(p9*2.830 ph21):f2

(p9*4.389 ph23):f2

(p9*2.722 ph23):f2

(p9*4.389 ph21):f2

(p9*2.778 ph23):f2

(p9*3.056 ph21):f2

(p9*0.333 ph23):f2

(p9*2.556 ph21):f2

(p9*4.000 ph23):f2

(p9*2.722 ph21):f2

(p9*4.111 ph23):f2

(p9*3.778 ph21):f2

(p9*3.889 ph23):f2

(p9*2.889 ph21):f2

(p9*3.000 ph23):f2

(p9*0.333 ph21):f2

(p9*2.500 ph23):f2

(p9*4.050 ph21):f2

(p9*2.830 ph23):f2

(p9*4.389 ph21):f2

(p9*2.722 ph23):f2

(p9*4.389 ph21):f2

(p9*2.778 ph23):f2

(p9*3.056 ph21):f2

(p9*0.333 ph23):f2

(p9*2.556 ph21):f2

(p9*4.000 ph23):f2

(p9*2.722 ph21):f2

(p9*4.111 ph23):f2

(p9*3.778 ph21):f2

(p9*3.889 ph23):f2

(p9*2.889 ph21):f2

(p9*3.000 ph23):f2

(p9*0.333 ph21):f2

(p9*2.500 ph23):f2

(p9*4.050 ph21):f2

(p9*2.830 ph23):f2

(p9*4.389 ph21):f2

(p9*2.722 ph21):f2

(p9*4.389 ph23):f2

(p9*2.778 ph21):f2

(p9*3.056 ph23):f2

(p9*0.333 ph21):f2

(p9*2.556 ph23):f2

(p9*4.000 ph21):f2

(p9*2.722 ph23):f2

(p9*4.111 ph21):f2

(p9*3.778 ph23):f2

(p9*3.889 ph21):f2

(p9*2.889 ph23):f2

(p9*3.000 ph21):f2

(p9*0.333 ph23):f2

(p9*2.500 ph21):f2

(p9*4.050 ph23):f2

(p9*2.830 ph21):f2

(p9*4.389 ph23):f2

lo to 9 times l1

;end DIPSI3

0.3u

#endif

if "l10 == 1" {

"d10 = in10 - 0.3u"

}

if "l10 > 0" {

(center (d10 cpds1):f1 (p3 ph20 p3*2 ph21 p3 ph20):f3)

0.3u do:f1

}

(p2 pl2 ph6):f2 (0.3u fq=cnst24):f1

;========water saturation=======================

4 (p20 pl20 ph20):f1

(p20 pl20 ph21):f1

(p20 pl20 ph22):f1

(p20 pl20 ph23):f1

(p20 pl20 ph23):f1

(p20 pl20 ph22):f1

(p20 pl20 ph21):f1

(p20 pl20 ph20):f1

lo to 4 times l2

;========end water saturation=======================

(p2 pl2 ph7):f2 (0.3u fq=0):f1

JCH2

(center (p1*2 pl1 ph0):f1 (p2*2 pl2 ph0):f2)

JCH2

(center (p1 pl1 ph8):f1 (p2 pl2 ph9):f2)

JCH1

(center (p1*2 pl1 ph10):f1 (p2*2 pl2 ph0):f2)

JCH1 pl29:f2

d16

go=2 ph31 cpds2:f2 finally do:f2 do:f1

1m do:f2 do:f1

10m mc #0 to 2

F1PH(calph(ph3, +90), caldel(d0, +in0) & calclc(l0,1))

F2PH(calph(ph6, -90), caldel(d10, +in10) & calclc(l10,1))

HaltAcqu, 1m

exit

ph0 = 0

ph1 = 0

ph2 = 3

ph3 = 0 2

ph6 = 1

ph7 = 3 3 1 1

ph8 = 0 0 0 0 2 2 2 2

ph9 = 2

ph10 = 0 2

ph11 = 2 0

ph20 = 0

ph21 = 1

ph22 = 2

ph23 = 3

ph31 = 0 2 2 0 2 0 0 2
